# Hyperbaric oxygen therapy as an immunosensitizing strategy in advanced gastric hepatoid adenocarcinoma: a case report

**DOI:** 10.3389/fimmu.2025.1625273

**Published:** 2025-06-27

**Authors:** Wenke Li, Jing Wei, Pengfei Zhang, Mo Cheng, Menghui Xu, Lin Zhu, Ming Liu

**Affiliations:** ^1^ Gastric Cancer Center/Cancer Center, West China Hospital, Sichuan University, Chengdu, Sichuan, China; ^2^ Department of Integrated Traditional and Western Medicine, West China Hospital, Sichuan University, Chengdu, China

**Keywords:** gastric cancer, hepatoid adenocarcinoma of the stomach, hyperbaric oxygen therapy, immune checkpoint inhibitors, immunosensitization, clinical complete response

## Abstract

**Background:**

Hepatoid adenocarcinoma of the stomach (HAS) is a rare but highly aggressive subtype of gastric cancer (GC) associated with an unfavorable prognosis, particularly in advanced or metastatic stages. While the standard first-line treatment for advanced GC involves immune checkpoint inhibitors (ICIs) combined with chemotherapy, HAS often shows a poor therapeutic response to this regimen. The hypoxia in the tumor microenvironment is considered a key factor limiting ICI efficacy, and combining hyperbaric oxygen therapy (HBOT) with immunotherapy may offer a synergistic sensitizing effect.

**Methods:**

We report a case of advanced HAS with peritoneal metastasis who received standard first-line immunochemotherapy (CAPOX plus sintilimab). After four cycles, the patient achieved only stable disease (SD) per RECIST 1.1 criteria. Consequently, HBOT was introduced as a sensitizing agent after the fifth cycle, and the patient subsequently completed the sixth cycle. This report was prepared using the CARE reporting guideline and checklist (Supplement A).

**Results:**

Following the addition of HBOT, the patient’s tumor markers normalized. Subsequent imaging and endoscopic evaluations revealed a complete resolution of all lesions, meeting the criteria for a clinical complete response (cCR) under RECIST 1.1.

**Conclusions:**

This case suggests that adding HBOT may enhance the efficacy of immunotherapy and overcome resistance to ICIs in advanced HAS. These promising findings warrant further investigation through prospective clinical studies to confirm this observation.

## Introduction

Hepatoid adenocarcinoma of the stomach (HAS), a rare subtype of gastric cancer (GC), accounts for only 0.3%-15% of all GC cases (1). Histologically, HAS resembles hepatocellular carcinoma, characterized by abundant eosinophilic cytoplasm, centrally located nuclei, and frequent elevation of serum alpha-fetoprotein (AFP). Clinically, HAS exhibits a high propensity for vascular invasion and distant metastasis, particularly to the liver and peritoneum, a prognosis that is even worse than that of conventional gastric adenocarcinomas (2).

The peritoneum is one of the most common metastatic sites in GC, occurring in approximately 4%–14% of patients (3). Treatment of gastric cancer with peritoneal metastasis (GCPM) remains challenging and primarily relies on systemic therapy. While the combination of chemotherapy and immune checkpoint inhibitors (ICIs) has improved survival outcomes in advanced GC, achieving a complete clinical response (cCR) in unresectable cases with peritoneal dissemination is exceedingly rare. Moreover, primary or acquired resistance to ICIs is common, significantly limiting their therapeutic efficacy. Recent preclinical studies have highlighted the potential of hyperbaric oxygen therapy (HBOT) to favorably modulate the tumor microenvironment (TME), alleviate tumor hypoxia, and enhance the efficacy of ICIs (4). Therefore, combining HBOT with standard chemotherapy and ICIs offers a promising strategy for immunosensitization or overcoming ICI resistance.

This report describes the case of a 62-year-old male patient with advanced stage IVB (rT2N2M1) HAS with peritoneal metastasis. Initial systemic therapy with CAPOX chemotherapy combined with sintilimab yielded limited efficacy. Remarkably, after the incorporation of HBOT into his regimen, the patient achieved a cCR. To our knowledge, this is the first reported case of advanced HAS with peritoneal metastasis achieving cCR with this combination, highlighting the potential of HBOT as a novel immunosensitizing therapeutic strategy in advanced GC.

## Case description

**Figure 1:**
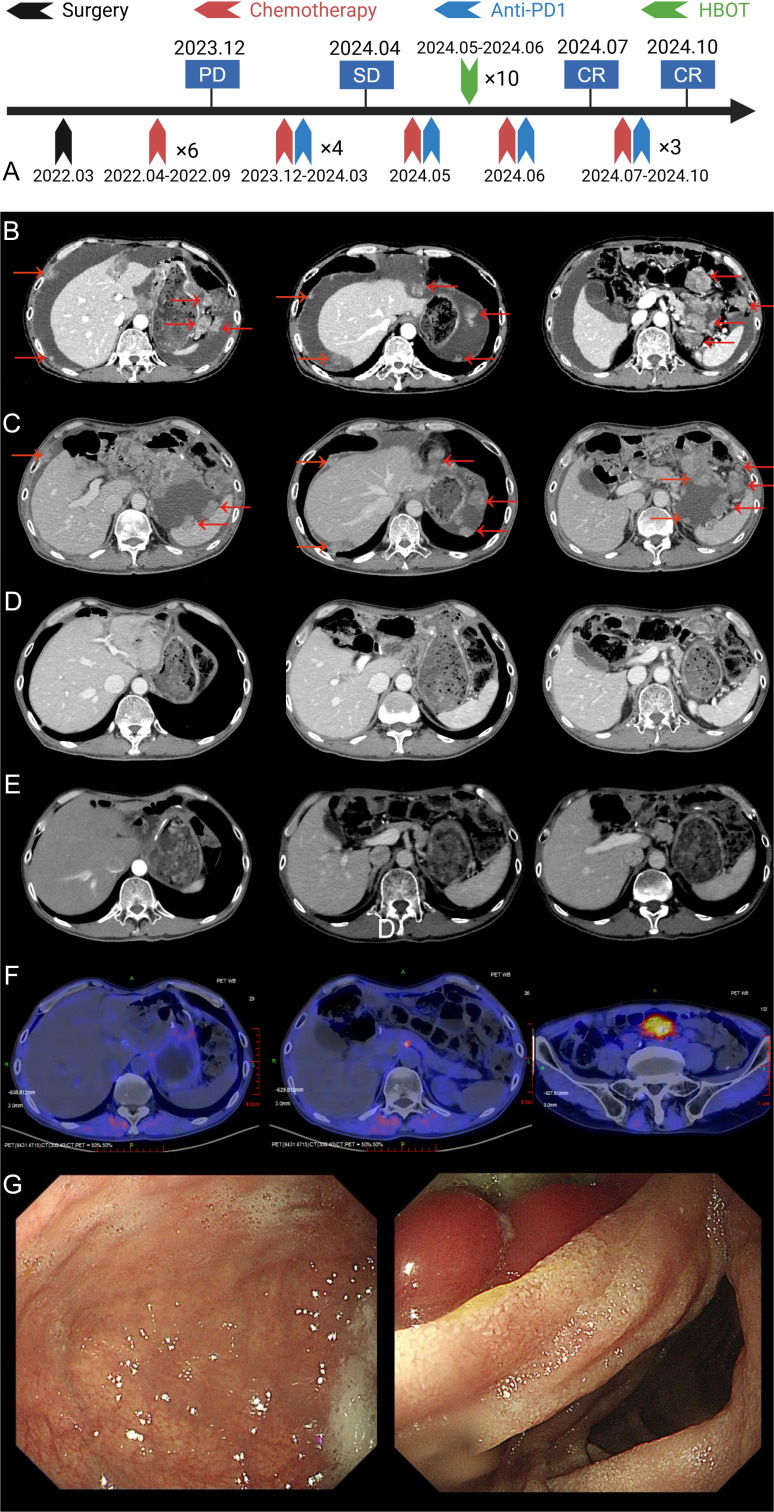
Imaging and treatment timeline of the patient. **(A)** The overall treatment timeline of the patient, including surgery, chemotherapy, anti-PD-1 therapy, and HBOT. **(B)** Abdominal enhanced CT at the time of recurrence showing tumor recurrence in the liver and surrounding tissues (tumor marked by red arrows). **(C)** Abdominal enhanced CT after 4 cycles of chemotherapy combined with ICIs treatment, showing partial tumor response (tumor marked by red arrows). **(D)** Abdominal enhanced CT after HBOT and an additional cycle of chemotherapy combined with ICIs, showing further tumor regression (tumor marked by red arrows). **(E)** Abdominal enhanced CT after 7–9 cycles of chemotherapy combined with ICIs, showing near-complete tumor resolution (tumor marked by red arrows). **(F)** FAPI PET/CT performed on January 3, 2025, revealing no evidence of tumor recurrence throughout the body. **(G)** Upper gastrointestinal endoscopy findings after 7–9 cycles of treatment. The gastric mucosa appeared smooth, and no active lesions were observed.

A timeline summarizing the key clinical events, from initial diagnosis to the last follow-up, is presented in **Figure 1A**.

### Patient background and initial diagnosis

**Figure 2:**
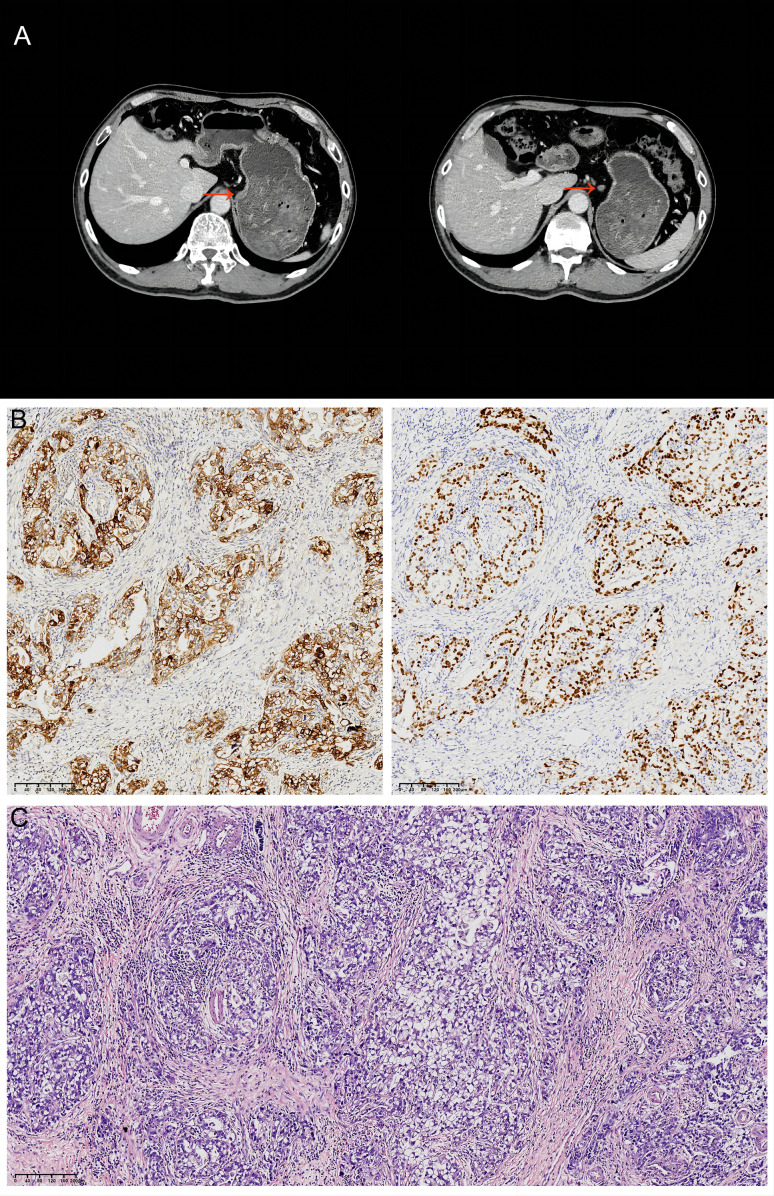
Initial CT findings and subsequent histopathology. **(A)** Abdominal CT from March 2022 showing thickening of the gastric lesser curvature (red arrows) and enlargement of the adjacent lymph nodes. **(B)** GPC3 (left) and SALL4 (right) immunohistochemical staining of the resected specimen. *Original magnification:×100; scale bar = 100 μm.* **(C)** Hematoxylin and eosin (H&E) staining of the resected specimen. *Original magnification:×100; scale bar = 100 μm*.

In March 2022, a 62-year-old male with no history of smoking, alcohol use, or other chronic diseases presented with epigastric pain. Psychosocially, the patient was a retired civil servant, married with strong family support, and had no history of significant psychological distress. His family history was notable for gastric cancer in his father, and subsequent genetic testing revealed a pathogenic RAD51D germline mutation alongside a TP53 somatic mutation of uncertain significance. Physical examination identified left upper quadrant tenderness, though laboratory results, including tumor markers and organ function tests, were within normal limits. An initial computed tomography (CT) scan demonstrated thickening of the gastric wall and enlarged perigastric lymph nodes (**Figure 2A**). Gastroscopy revealed ulcers at the gastric angle, and biopsy confirmed moderately to poorly differentiated adenocarcinoma. Based on these findings, the patient was clinically staged as cT2N1M0 (Stage IIA) according to the AJCC 8th Edition Cancer Staging Manual.

### Surgical treatment and pathological findings

On March 16, 2022, the patient underwent laparoscopic radical distal gastrectomy with D2 lymphadenectomy, achieving a complete (R0) resection. Postoperative pathological examination of the resected specimen revealed a 4.8 × 4.0 cm, Borrmann type II tumor with a rough surface, which had focally infiltrated the muscularis propria. Histologically, the tumor was classified as an intestinal-type adenocarcinoma, that was predominantly tubular and graded as G2-G3 (moderately to poorly differentiated). Metastatic carcinoma was identified in three lymph nodes, one each from stations 3a, 7, and 11P.

Immunohistochemistry (IHC) analysis yielded the following results: HER2 (1+), CLDN18.2 (0), PD-L1 (CPS=2), and proficient mismatch repair (pMMR). PD-1 was positive in a minority of lymphocytes, while CDX2 and CK20 were positive, and Desmin was positive in smooth muscle. Epstein-Barr virus-encoded small RNA (EBER) *in situ* hybridization was negative. Additional IHC markers revealed positivity for SALL4 and GPC3, and negativity for CD34, AFP, and SOX10. These findings led to a final diagnosis of adenocarcinoma with features of both enteroblastic differentiation and hepatoid adenocarcinoma (**Figures 2B, C**). Based on the AJCC 8th Edition TNM staging system, the pathological stage was confirmed as pT2N2M0, Stage IIA.

### Postoperative adjuvant chemotherapy and recurrence

Following surgery, the patient completed six cycles of adjuvant SOX chemotherapy and entered a period of surveillance. However, in December 2023, approximately one year after his initial surgery, he presented with abdominal distension. Laboratory tests revealed markedly elevated levels of serum AFP (>1210 ng/ml) and CA724 (16.10 U/ml). An abdominal CT scan subsequently confirmed the presence of peritoneal metastasis (**Figure 1B**). To establish a definitive diagnosis of recurrence, a therapeutic and diagnostic paracentesis was performed on December 8, 2023, which yielded approximately 700 mL of hemorrhagic ascitic fluid. Cytological analysis of this fluid confirmed recurrent adenocarcinoma. Consequently, the patient’s diagnosis was updated to recurrent hepatoid adenocarcinoma of the gastric angle with peritoneal metastasis, and he was restaged as rT2N2M1 (Stage IV) according to the AJCC staging system.

### Systemic therapy

Following the diagnosis of recurrence, the patient received a single dose of intraperitoneal paclitaxel (60 mg) on December 9, 2023. Subsequently, from December 10, 2023, to March 12, 2024, he was treated with four cycles of first-line systemic immunochemotherapy. This regimen, administered every three weeks, consisted of CAPOX (capecitabine: 1.5 g orally, twice daily on days 1–14; oxaliplatin: 200 mg by intravenous infusion on day 1) combined with sintilimab (200 mg by intravenous infusion on day 1). An initial treatment response evaluation was performed in March 2024. An enhanced abdominal CT scan (**Figure 1C**) revealed stable disease (SD) according to the RECIST 1.1 criteria.

### Effective combination therapy

The decision to add HBOT was prompted by the limited clinical benefit observed after the initial four cycles: imaging evaluation revealed stable disease (SD), and the patient’s abdominal distension had not significantly improved. To overcome potential resistance and prevent disease progression, and because the evaluation coincided with the scheduled start of the fifth cycle, the clinical team decided to introduce HBOT as a sensitizing strategy immediately after administering the next planned cycle of immunochemotherapy to maintain treatment continuity. Accordingly, on April 30, 2024, the patient received the 5th cycle of CAPOX and sintilimab with all drug doses maintained as in previous cycles. This was immediately followed by 10 sessions of HBOT (1-hour oxygen inhalation at 2 atmospheres absolute [ATA] per session). After completing the sixth cycle of the combined therapy on June 22, 2024, a follow-up evaluation in July 2024 showed a remarkable response. An enhanced abdominal CT scan revealed a complete resolution of the recurrent lesion at the gastric anastomosis and the peritoneal metastases (**Figure 1D**). Concurrently, tumor markers normalized, with AFP decreasing to 3.06 ng/mL (normal range: 0–7 ng/mL) and CA724 to 2.81 U/mL (normal range: 0–6.9 U/mL). The changes in AFP during treatment are illustrated in the **Table 1**. Based on RECIST 1.1 criteria, the patient had achieved complete response (CR).

**Table 1:** Changes in AFP levels during treatment.

Date	AFP (ng/ml)
2022-03-03	2.57
2023-12-08	>1210.00
2024-01-03	>1210.00
2024-02-06	>1210.00
2024-03-12	>1210.00
2024-04-30	>1210.00
2024-07-17	3.06
2024-10-14	1.72
2024-11-14	1.88

The patient then proceeded to maintenance therapy with capecitabine and sintilimab. The maintenance regimen included the following: capecitabine: 1.5 g orally, twice daily, from day 1 to day 14; sintilimab: 200 mg by intravenous infusion, day 1. Each cycle was administered every three weeks. Follow-up imaging, including an enhanced chest and abdominal CT scan on October 15, 2024, confirmed a sustained cCR (**Figure 1E**). A fibroblast activation protein inhibitor (FAPI) PET/CT scan on January 3, 2025, showed a focal area of high tracer uptake in the peritoneum without a corresponding mass on CT, a finding suggestive of post-treatment inflammation rather than tumor recurrence (**Figure 1F**). Finally, a painless endoscopy on November 18, 2024, revealed no abnormalities in the remnant stomach (**Figure 1G**), further corroborating the complete response.

### Patient perspective

The patient reported no increase in adverse effects after the addition of hyperbaric oxygen therapy and demonstrated good treatment compliance throughout the course.

### Follow-up

The patient is currently undergoing follow-up, including hematological tests, imaging evaluations, and quality of life assessments. Compliance with the treatment plan is excellent, and the patient maintains a good quality of life, with no signs of recurrence or significant adverse effects observed. As of the last follow-up on March 29, 2025, the patient remained progression-free. The progression-free survival (PFS) since the confirmation of recurrence on December 8, 2023, has been ongoing for over 15 months.

## Discussion

The management of HAS, a rare but highly aggressive subtype of GC, presents a significant clinical challenge due to its poor prognosis and limited response to standard therapies. Characterized by histological features resembling hepatocellular carcinoma, HAS is frequently associated with elevated serum AFP levels (2, 5). Despite its rarity, the high global burden of GC, one of the top five most common malignancies worldwide, means that a substantial number of patients are still diagnosed with HAS each year (6). Currently, the therapeutic strategies for HAS do not differ from those for conventional gastric adenocarcinoma. For patients with unresectable advanced or metastatic HAS, systemic therapy remains the primary treatment modality. Pivotal Phase III trials, such as KEYNOTE-859 (7), CheckMate-649 (8), and ORIENT-16 (9), have established the combination of chemotherapy and a PD-1 inhibitor as the standard of care for first-line treatment of HER2-negative advanced GC (with mPFS of 6.9–7.7 months), a recommendation endorsed by major clinical guidelines including NCCN, CSCO, and ESMO. Indeed, subsequent meta-analyses have confirmed that this combination provides a significant survival benefit compared to chemotherapy alone (10). However, HAS is associated with a poorer prognosis than conventional GC, which is attributed to its higher propensity for distant metastasis and limited responsiveness to immunotherapy (11). Consequently, developing strategies to overcome this resistance and sensitize HAS to immunotherapy represents a critical clinical challenge.

The present case offers evidence for the potential of HBOT. The patient, diagnosed with advanced HAS and peritoneal metastasis, initially showed a suboptimal response to four cycles of standard immunochemotherapy (CAPOX plus sintilimab), achieving only SD. However, a dramatic clinical turnaround was observed following the introduction of HBOT after the fifth cycle. Subsequent evaluations revealed a cCR, evidenced by the normalization of tumor markers and the disappearance of all lesions on imaging and endoscopic examinations. Notably, the patient’s progression-free survival has already exceeded 15 months. This remarkable improvement strongly suggests that HBOT acted as a potent immunosensitizing agent, overcoming the initial resistance and unlocking the therapeutic potential of the existing immunotherapy regimen.

The poor responsiveness of HAS to immunotherapy is largely attributed to its unique TME. HAS is typically characterized as an immunologically “cold” tumor, featuring diminished infiltration of CD8+ T cells, an abundance of regulatory T cells (Tregs) and M2-type macrophages, low PD-L1 expression, and a low tumor mutational burden (TMB). The majority of HAS cases are also microsatellite stable (MSS), all of which are hallmarks of poor responsiveness to immunotherapy (12). A salient feature of HAS is the elevation of serum AFP (11), a biomarker strongly associated with poor prognosis in GC. Elevated AFP not only indicates a poor outcome but also actively promotes tumor progression by enhancing proliferation, invasion, and migration (13). Furthermore, a distinctive feature of HAS is its extensive and abnormal tumor vasculature, which has been linked to the overexpression of vascular endothelial growth factor C (VEGF-C) and angiopoietin-like proteins (ANGPTLs) (14, 15). AFP itself can exacerbate this by upregulating VEGF expression and increasing microvessel density (14). However, this dysregulated angiogenesis results in dysfunctional vessels that are irregular, tortuous, and poorly branched. The consequent inefficient blood perfusion leads to extensive hypoxic regions within the tumor, creating a profoundly hypoxic and immunosuppressive TME (16). This tumor-induced hypoxia is a pivotal factor undermining the efficacy of ICIs. It triggers a cascade of immunosuppressive events, primarily through the activation of the hypoxia-inducible factor-1α (HIF-1α) signaling pathway. Activation of HIF-1α promotes the recruitment of inhibitory cells, including myeloid-derived suppressor cells (MDSCs), Tregs, and M2-type tumor-associated macrophages. It also upregulates PD-L1 expression on both cancer and dendritic cells, which directly inhibits cytotoxic T lymphocyte function and reinforces the immunosuppressive landscape (17–21). Additionally, hypoxic conditions are known to promote cancer stem cell maintenance, thereby increasing tumor invasiveness and therapeutic resistance (22, 23). Collectively, these features likely explain the limited efficacy of the initial immunochemotherapy regimen observed in our patient.

HBOT may enhance the efficacy of ICIs through multiple synergistic mechanisms. Foremost, HBOT directly counteracts the hypoxic tumor microenvironment. By increasing oxygen tension, it downregulates the expression of HIF-1α, thereby mitigating the recruitment of immunosuppressive cells such as Tregs and M2-macrophages (24–26). Concurrently, the hyperoxic state promotes the normalization of tumor vasculature, partly by upregulating the expression of platelet endothelial cell adhesion molecule-1 (PECAM-1/CD31), which facilitates increased infiltration of immune cells into the tumor site (27). Beyond remodeling the TME, HBOT has been shown to directly augment the effector functions of immune cells. It can enhance the cytotoxic activity of both effector T cells and natural killer (NK) cells, boosting their antitumor capabilities (27). Furthermore, emerging evidence suggests that HBOT can amplify the efficacy of PD-1 blockade by activating the cGAS-STING signaling pathway, a key innate immune sensing mechanism (27). Another proposed mechanism involves the degradation of the extracellular matrix (ECM) by HBOT, which may improve the physical delivery and penetration of large-molecule therapeutics like anti-PD-1/PD-L1 monoclonal antibodies into the TME (28). Collectively, these multifaceted mechanisms provide a strong theoretical basis for the role of HBOT as an effective immunosensitizing strategy in cancer therapy.

Indeed, the “abnormal vasculature-hypoxia-immunosuppression axis” is not exclusive to HAS but is a common pathological feature of gastric adenocarcinoma, particularly in advanced stages (16, 29). This hypoxia-driven, immunosuppressive microenvironment is thought to be a key mechanism underlying the limited efficacy of ICIs in a significant proportion of GC patients. Consequently, this highlights the therapeutic potential of HBOT in the broader population of patients with advanced or metastatic gastric adenocarcinoma. Upon reviewing the existing clinical literature, we found that studies on HBOT in cancer treatment are limited, with most focusing on its role as an adjunct to radiotherapy or chemotherapy, primarily aimed at improving quality of life, and demonstrating good tolerability in most cases. To further investigate the immunosensitizing mechanisms of HBOT and to validate the efficacy of this combination therapy, our team has initiated a Phase Ib/II clinical trial. This study will evaluate CAPOX chemotherapy combined with the PD-1 inhibitor sintilimab and HBOT as a first-line treatment for advanced or metastatic gastric or gastroesophageal junction cancer (GC/GEJC) (NCT06742411).

To our knowledge, this is the first reported case of a patient with advanced, HER2-negative HAS and peritoneal metastasis achieving a cCR through the combination of HBOT with chemotherapy and immunotherapy. This outcome highlights the potential of HBOT as an effective immunosensitizing strategy in GC. The primary strengths of this report lie in its novelty, as well as the favorable safety profile and low cost of HBOT, which would facilitate its widespread adoption if proven effective in large-scale clinical trials. However, this case report has several limitations. Firstly, as a single case, it lacks a control group for comparison, and the influence of the patient’s specific, albeit of uncertain significance, genetic mutations on the observed efficacy of HBOT and ICIs remains unknown. Secondly, because no residual tumor was found during the follow-up endoscopy, we were unable to perform a comparative analysis of the TME before and after HBOT to elucidate the underlying mechanisms. Furthermore, predictive biomarkers to identify patients who would most benefit from the HBOT-immunochemotherapy combination are currently lacking. Although imaging techniques to assess tumor hypoxia in GC exist, their accuracy and correlation with treatment outcomes need to be validated in larger-scale clinical trials. Therefore, despite the promising results of this case, further prospective studies are essential to confirm these findings, explore the mechanisms of action, and define the optimal patient population for this novel therapeutic approach.

## Conclusion

This case report details the successful treatment of a patient with advanced HAS who achieved a remarkable clinical CR. The addition of HBOT to a standard immunochemotherapy regimen appeared to overcome initial treatment resistance. These findings suggest that HBOT may serve as an effective immunosensitizing strategy, enhancing the efficacy of ICIs in the treatment of GC. Future clinical studies are warranted to further verify the efficacy and safety of HBOT combined with immunotherapy, explore its mechanisms of action, and provide new insights for the comprehensive treatment of advanced GC.

## Data Availability

The original contributions presented in the study are included in the article/supplementary material. Further inquiries can be directed to the corresponding authors.
